# The effect of biogenic selenium nanoparticles (*Cissus quadrangularis*) on the performance and antioxidant status of piglets in the climatic conditions of Zambia

**DOI:** 10.3389/fvets.2025.1742829

**Published:** 2026-02-02

**Authors:** Pompido Chilala, Sylvie Skalickova, Pavel Horky

**Affiliations:** Department of Animal Nutrition and Forage Production, Faculty of AgriSciences, Mendel University in Brno, Brno, Czechia

**Keywords:** *Cissus quadrangularis*, climate resilience, nanoparticles, piglets, selenium

## Abstract

Thermal stress limits sustainable pig production in tropical regions by impairing growth, feed efficiency, and welfare. This study evaluated dietary *Cissus quadrangularis* selenium nanoparticles (CQ-SeNPs) for supporting growth performance and antioxidant capacity in pigs under natural tropical conditions in Zambia. Thirty-three weaner pigs were randomly assigned to three diets: control (basal), inorganic selenium (Na₂SeO₃), or CQ-SeNPs, and monitored for 30 days under moderate ambient temperatures below heat stress thresholds (27–32 °C for lighter and 18–28 °C for heavier piglets). Growth performance, including body weight, average daily gain (ADG), and feed conversion ratio (FCR), was measured. CQ-SeNPs supplemented pigs showed numerically higher ADG (267 g/day) and lower FCR (1.98) compared with control (264 g/day, 1.99) and Na₂SeO₃ (253 g/day, 2.04), but differences were not statistically significant (*p* > 0.05). Body weight trends were similar across treatments, indicating limited activation of stress-related physiological pathways under these conditions. These results indicate that CQ-SeNPs supplementation is safe and may support growth under oxidative or thermal challenge, though further studies under defined heat stress are needed to confirm efficacy. Overall, findings highlight the potential of phytogenic selenium nanoparticles in developing climate-adaptive feeding strategies for sustainable swine production in tropical regions.

## 1 Introduction

The swine production sector plays a pivotal role in many farming systems around the world in meeting food requirements; nevertheless, the swine industry is faced with numerous challenges (1). The most prevalent challenges are environmental stresses that affect the health, welfare and productivity of animals (2). In recent years, thermal stress has been observed to pose significant problems to the swine industry across the world (3). Countries possessing tropical and subtropical climates experience higher ambient temperatures which affect the productivity of weaner pigs whose thermoneutral zone for fattening is between 26 and 32 °C (4, 5). Swine are known to be highly sensitive to high ambient temperatures as a result of having limited abilities to sweat (6). This has been observed to result in reduced feed intake, low weight gain, poor meat quality and reproductive problems (3, 6). The difficulties posed by high temperatures to swine production necessitate the need to develop effective strategies to combat heat stress during periods of adverse weather conditions (7, 8).

*Cissus quadrangularis* (CQ) is a promising medicinal plant for mitigating the effects of heat stress due to the presence of important bioactive compounds responsible for enhancing antioxidant defence in animals (9, 10). CQ is a succulent plant of the family Vitaceae, native to tropical Africa and Asia; it is widely distributed and used in Africa for its beneficial properties (10). CQ has been used as a traditional medicine for livestock and humans by native populations of Africa for many years (11–13). According to Dhanasekaran (14), CQ possess antioxidant and anticancer properties that can be crucial in mitigating the effects of heat stress faced by swine farmers in the tropics. Furthermore, CQ has important natural bioactive compounds such as carotenoids, triterpenoids and ascorbic acid with antioxidant and anti-inflammatory properties which may have beneficial effects against heat stress (14) According to Sundaran et al. (15), CQ plant extracts have strong antioxidant and free radical scavenging activities as a result of having β-carotene. The plant stems are rich in polyphenols, flavonoids and other bioactive compounds that show potent antioxidant activities against oxidative stress (16). Our novel approach in this research is the application of CQ in pig nutrition as an antioxidant, which remains largely unexplored.

Biogenic selenium nanoparticles (SeNPs) have gained attention in science as effective antioxidants due to their enhanced bioavailability, antioxidant capacity and effectiveness (17, 18). They are also less toxic and more environmentally friendly compared to inorganic selenium forms (19). The main component of SeNPs is selenium, a widely studied trace element that is crucial for immune functions and antioxidant defense mechanisms in animals and plants (20). Biogenic SeNPs have been found to exhibit better absorption and utilization rates in animals, making them a potent tool for mitigating the effects of heat stress in swine (19). Various research findings have concluded that SeNPs enhance antioxidant enzyme activities and improve thermotolerance in various livestock species including pigs exposed to high ambient temperatures (19, 21). This makes biogenic SeNPs an important tool for alleviating effects of heat stress in pigs under Zambian conditions.

Despite these well documented benefits of CQ and biogenic SeNPs, their combined effect in alleviating heat stress in pigs has not been explored. Understanding the synergistic effects of this combination is vital in enhancing pig production during periods of high ambient temperatures in tropical climates such as Zambia. This study provides novel scientific knowledge on the efficacy of CQ-biogenic SeNPs in mitigating the effects of heat stress in pigs under tropical Zambian conditions. The study also evaluates the growth performance of pigs under heat stress including parameters such as antioxidant activity and serum total protein. The research outcomes offer important information on feed additives that can be used to alleviate heat stress in tropical climates.

## 2 Materials and methods

### 2.1 Experimental design

This study was conducted in accordance with the guidelines of the Helsinki declaration and approved by the international review board (ethics committee) a commission that ensures protection of the welfare of experimental animals of Mendel University in Brno (protocol code 16OZ27083/2014-17,214 approved on 20th May 2019). A total of 33 Topigs Norsvin (TN70) weaned piglets of similar weight were selected for this experiment. The mean body weights of the 33 pigs were 7.85 ± 1.55 kg at the beginning of the trial, three groups of 11 piglets each were established. The piglets were observed and fed on experimental rations for 30 days, the first group was the control (control—Ctrl) fed on normal rations without any additives. The second group was a positive control (control—Na_2_SeO_3_) with Sodium selenite (Na_2_SeO_3_) added to feed rations at 0.5 mg of Se/kg, The third group was the research group (treatment—CQ-SeNPs) which was fed on a ration consisting of Nanoparticle (NPs) synthesized from *Cissus quadrangularis(CQ)* in combination with biogenic selenium at 0.5 mg Se/kg inclusion to a feed ration. A dose of 0.5 mg of Se/kg was chosen in this experiment due to the narrow toxicity range of selenium in animals. CQ plants were collected from Kasisi Agricultural Training Centre and identified by a botanist from the Copperbelt University, with the vegetative growth stage recorded at the time of collection. Voucher specimens were prepared for future reference (22).

The Na_2_SeO_3_ and NPs were mixed consistently with the feed rations for each treatment every day to ensure accurate results. The control feed rations were also mixed in the same way without any additions to ensure uniformity in treatment. Feed rations of the three groups were weighed with a digital scale to ensure the quantities were uniform and of the required amounts every day. In accordance with the requirements of the fattening period of Zambian pigs, the environmental conditions were similar in all the rooms under observation. All the pigs were fed on a standard diet of weaner feed at a dose of 0.4 kg/pig per day. The composition and nutritional values of the weaner feed are shown in Table 1. These diets were compiled in accordance with TN70 feed requirements by Zambia Pig Genetics (ZPG) the main suppliers of the TN70 pig breed in Zambia. All animals under this experiment had ad libitum access to feed and water for drinking and were closely monitored for any abnormalities.

**Table 1:** Proximate composition of the samples (%).

Analysis name	Units	Result
Moisture	%	9.81
Protein	%	19.23
Fat	%	5.18
Fibre	%	4.31
Ash	%	5.51

### 2.2 Housing and temperature measurements

The pigs under this experiment were housed in 3 identical pens of 4 by 3 meters each and had access to water throughout the research. Room temperature measurements were conducted every 3 h using a temperature datalogger (Elitech RC-5, UK). The datalogger was calibrated before the experiment using a two-point check against a NIST-traceable thermometer in an ice bath (0 °C) and a 40 °C warm bath, following the manufacturer’s ± 0.5 °C accuracy specification. Recorded offsets were within acceptable limits, and weekly single-point ambient verifications were performed throughout the study.

The logger was enclosed in a polyethylene cover for protection from dust and moisture.

**Figure 1:**
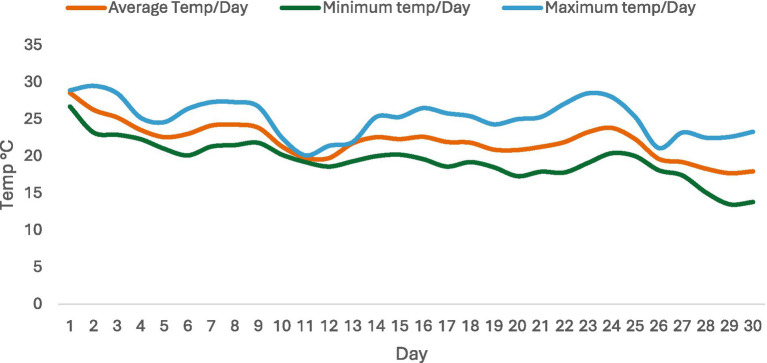
Daily ambient temperature readings in pig pens over a 30-day period.

The logger was placed at a height of 1.5 meters above the internal floor within rooms under observation to obtain accurate readings throughout the experiment. The datalogger temperature measurement range was −30 to 70 °C, with an accuracy of ±0.5 °C (−20 °C~+40 °C); others, +1 °C and an error margin of ±0.5 °C. Ambient temperature measurements are shown in Figure 1.

### 2.3 Feed ration analysis

Feed samples were collected and oven dried at temperatures of up to 50 °C and ground to smaller particles to increase the surface area of the particles to sizes of 1 mm. The ground particles were analysed for basic nutrient contents to ensure that all inconsistencies in growth are not attributed to feed rations. Fibre was analysed using an A200 fibre analyser (ANKOM, Czechia) and the analysis of nitrogenous substances was conducted according to the Kjeldahl method (N x 6.25) based on the AOAC official method 2001.11. Fat content analysis was conducted using direct extraction in accordance with the Soxhlet method in accordance with AOAC official method 920.39. Ash content analysis was conducted using a calorimeter (IKA C5000 Werke, Germany) where samples were burnt for 4.5 h in an oven at a set temperature of 550 °C. Consistent with AOAC official method 942.05, which outlines the standard procedure for total ash determination in animal feeds (23–25).

### 2.4 Chemicals

Na2SeO3, ethanol, ABTS, potassium persulphate, and other chemicals unless noted otherwise were purchased from Sigma Aldrich (USA). CQ plant leaves were collected from the forest and dried at room temperature in Zambia. The pH value of pig blood serum was measured using inoLab Level 3 (Wissenschaftlich-Technische Werkstatten GmbH; Weilheim, Germany). Deionised water underwent demineralization by reverse osmosis using the instruments Aqua Osmotic 02 (Aqua Osmotic, Tisnov, Czech Republic).

### 2.5 *Cissus quadrangularis* SeNPs synthesis

One gram of CQ plant powder was dissolved in 10 mL of dH2O. The plant sample was incubated at 22 °C and 60 °C (Heating chamber, Binder, Germany) for 1 h or 24 h with occasional shaking by hand. After incubation, the sample was centrifuged (MPW-350e, Poland), supernatants were collected and filtered via a 0.2 μm nylon syringe filter (CHS Filterpure, Chromservis, Czechia). One mL of plant extract was slowly added to a 9 mL solution of sodium selenite (10 mM) under continuous stirring on a magnetic stirrer. The mixture was covered with parafilm and allowed to react at 22 °C, 600 rpm, for 24 h. SeNPs were stored at 4 °C.

Characterization of the synthesized CQ–SeNPs has been previously reported in detail in our earlier publication (22), were TEM analysis confirmed predominantly spherical nanoparticles with an average size of ~40–80 nm, while DLS measurements showed a hydrodynamic diameter consistent with stable colloidal dispersion. The zeta potential values (approximately −25 to −30 mV) indicated good electrostatic stability, and the morphology remained uniform with no significant aggregation. These physicochemical properties support the suitability of the CQ–SeNPs for biological applications and justify their use in the present study.

### 2.6 Blood analysis

At 15 and 30 days, blood samples were collected from all piglets under observation. Blood samples were extracted from the external jugular vein of each piglet using a 5 mL syringe and an18-gauge needle. Samples were stored in 4 mL serum tubes for transportation to the lab and were centrifuged at 4,000 rpm using a laboratory medical low speed centrifuge (LC-04A). Serum was analysed within 1 week of collection to ensure sample integrity and accuracy of the results. The main aim of collecting samples was to determine haematological and biochemical parameters. A Konelab T20xt Biochemical analyser (Thermo Fischer Scientific) was used for spectrometric analysis using commercially available reagents according to a method elaborated by Horky et al. (26). Samples were further monitored for activities of Glutathione peroxidase (GPx) and Superoxide dismutase (SOD) based on elaborated methods by Urbankova et al. (27). Determination of SOD and GPx colorimetric assay according to a protocol were based on the assay manufacturer (Elabscience, USA).

### 2.7 Description of experimental data

The statistical analysis was performed to assess the effects of treatments on piglets under heat stress conditions. Assumptions of normality and homogeneity of variance were checked prior to analysis. Data was analysed using repeated measures ANOVA to account for temporal changes over the 30-day experimental period. At the start of the experiment, the average weight of piglets in the different rooms was 7.85 ± 1.55 kg, and their age was 40 days. No mortality was recorded during the period of experimentation. The collected data was interpreted using STATISTICA. CZ and Microsoft excel. Statistical analysis was performed using repeated measures ANOVA to account for measurements overtime.

## 3 Results

### 3.1 Growth and efficiency parameters

Swine supplemented with CQ-SeNPs exhibited the highest average daily gain (ADG) of 267grams/day followed by the C at 264 grams/day and 253 grams/day for Na_2_SeO_3_. The lowest feed conversion ratio (FCR) was also observed in the CQ-SeNPs group at 1.98 indicating slightly improved growth performance and feed efficiency compared to the C and the Na_2_SeO_3_ groups. Despite the observed differences, there were no statistically significant variations in body weights among the groups under observation (see Table 2).

**Table 2:** Comparisons of the effects of Na_2_SeO_3_, CQ-SeNPs and the control on growth performance in pigs over 30 days.

Parameter	Unit	Control mean ± SD	Na₂SeO₃ mean ± SD	CQ-SeNPs mean ± SD	*p*-value
Initial body weight (0 days)	kg	7.83 ± 1.23	7.86 ± 1.94	7.85 ± 1.41	0.998
Body weight (15 days)	kg	9.33 ± 1.68	9.50 ± 1.95	9.76 ± 1.70	0.982
Body weight (30 days)	kg	16.01 ± 2.73	15.85 ± 3.79	16.64 ± 3.17	0.931
Total weight gain	kg	8.18 ± 2.12	7.99 ± 2.40	8.79 ± 2.30	0.941
Average daily gain (ADG)	g/day	264 ± 12	253 ± 14	267 ± 10	0.926
Feed conversion ratio (FCR)	–	1.99 ± 0.04	2.04 ± 0.05	1.98 ± 0.03	0.911

CQ SeNPs = *Cissus quadrangularis* selenium nanoparticles, Control = Diet without supplementation, Na_2_SeO_3_ = Inorganic Sodium selenite. Mean ± SD (*n* = 11 pigs per group). Statistical analysis was performed using repeated measures ANOVA to account for measurements over time. No significant differences were observed among treatments or time points (*p* > 0.05).

### 3.2 Analysis of oxidative stress, antioxidant biomarkers and protein quantification

#### 3.2.1 Serum total protein

**Figure 2:**
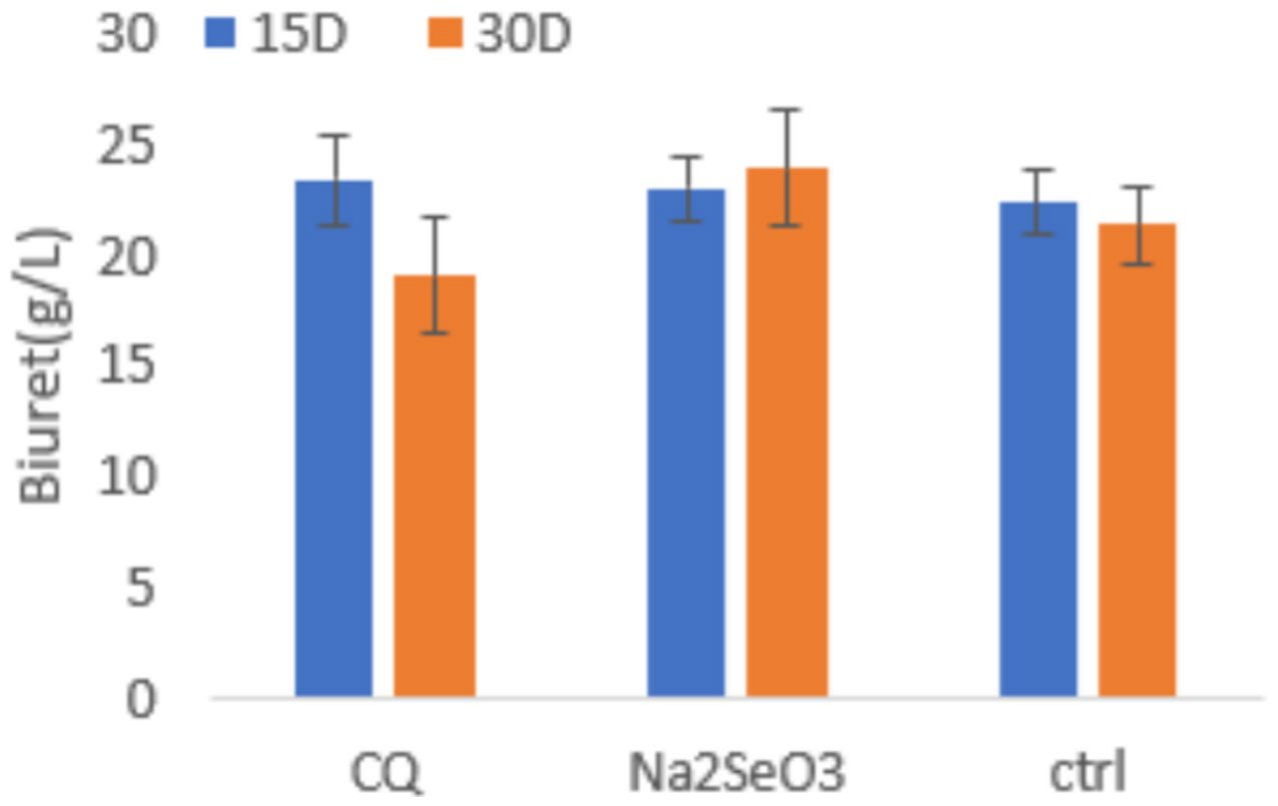
Serum total protein in weaner pigs under three dietary treatments: CQ (*Cissus quadrangularis* selenium nanoparticles), Na_2_SeO_3_ (inorganic sodium selenite), and Ctrl (control diet without additives) at 15 and 30 days. Data are presented as mean ± SD (*n* = 11 pigs per group). Blue bars represent 15 days, orange bars represent 30 days. Statistical analysis was performed using repeated measures ANOVA to account for measurements over time.

According to our biuret assay results (Figure 2), total protein contents were similar among all dietary groups at 15 days. At 30 days, the Na₂SeO₃ group exhibited the highest protein contents followed by the C group, while CQ-SeNPs showed the lowest content.

### 3.3 Antioxidant enzyme activities

**Figure 3:**
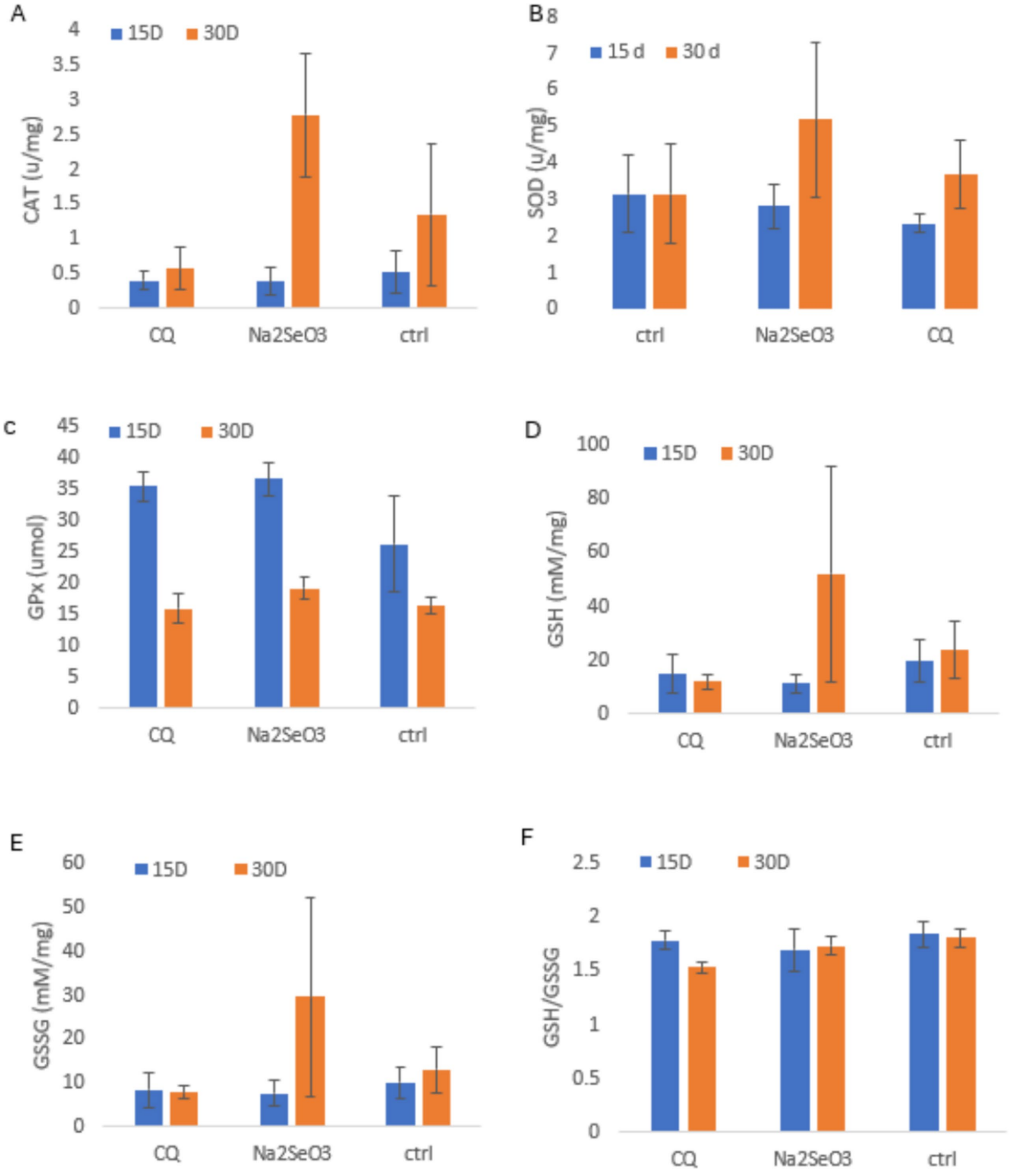
Shows antioxidant enzyme activities and oxidative stress markers in piglets fed CQ (*Cissus quadrangularis*), Na_2_SeO_3_ (inorganic sodium selenite) and Ctrl (control diet without any additives) at 15 and 30 days. Data are mean ± SD (*n* = 11 pigs per group); blue = 15 days, orange = 30 days. Panels: **(A)** Catalase (CAT, U/mg); **(B)** Superoxide dismutase (SOD, U/mg); **(C)** Glutathione peroxidase (GPx, umol); **(D)** Reduced glutathione (GSH, mM/mg); **(E)** Oxidized glutathione (GSSG, mM/mg); **(F)** GSH/GSSG ratio. Statistical analysis was performed using repeated measures ANOVA to account for measurements over time.

Antioxidant enzyme activities showed clear time-dependent adaptation to supplementation (Figures 3A–F). By day 30, Na₂SeO₃ produced the highest CAT, SOD, GSH and GSSG levels, reflecting a delayed but stronger antioxidant response consistent with the gradual upregulation of selenoproteins. CQ-SeNPs induced earlier but weaker and less sustained enzyme activity GPx increased similarly in both supplemented groups at day 15, aligning with the faster responsiveness of GPx to available selenium, but Na₂SeO₃ maintained slightly greater activity by day 30. Oxidative balance markers followed the same pattern, Na₂SeO₃ showed enhanced late-phase adaptation, while CQ-SeNPs had the lowest GSH/GSSG ratio. Although differences were not statistically significant (*p* > 0.05), the temporal trends indicate stronger overall antioxidant adaptation with Na₂SeO₃.

### 3.4 Total antioxidants capacity, oxidative stress and heat shock markers

**Figure 4:**
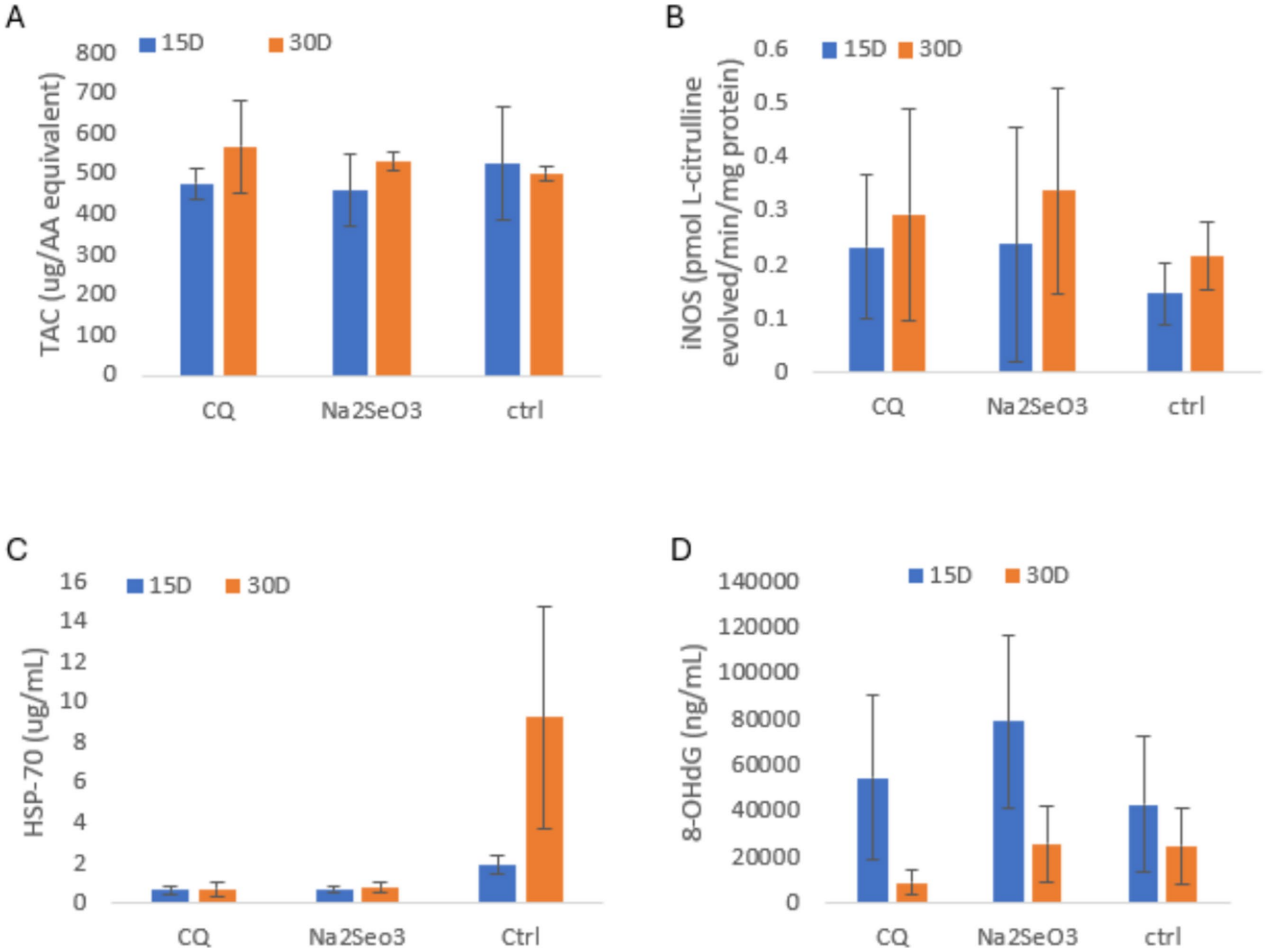
Shows antioxidant, inducible nitric oxide synthase, heat shock, and oxidative DNA damage markers in pigs fed CQ (*Cissus quadrangularis*), Na_2_SeO_3_ (inorganic sodium selenite) and Ctrl (control diet without any additives) at 15 and 30 days. Data are mean ± SD; blue = 15 days, orange = 30 days. Panels: **(A)** Total antioxidant capacity (TAC, μg AA equivalent); **(B)** Inducible nitric oxide synthase activity (iNOS, pmol L-citrulline/total protein/mg protein); **(C)** Heat shock protein 70 (HSP-70, μg/mL); **(D)** 8-hydroxy-2′-deoxyguanosine (8-OHdG, ng/mL).

As shown in Figures 4A–D, TAC was slightly higher in the C at 15 days, while CQ-SeNPs showed the highest activity at 30 days. iNOS levels were comparable across groups at 15 days but Na₂SeO₃ exhibited the highest results at 30 days. 8-OHdG was elevated in Na₂SeO₃ at 15 days, but CQ-SeNPs showed the lowest levels at 30 days, suggesting reduced DNA oxidation. HSP-70 remained low in treated groups, while the control showed a marked increase at 30 days, indicating greater cellular stress. No significant differences were observed (*p* > 0.05).

## 4 Discussion

In this study, pigs supplemented with a diet consisting of CQ-SeNPs exhibited slightly improved growth performance and feed efficiency compared with those receiving Na₂SeO₃ or the C diet. However, these differences were not statistically significant. This is as a result of the experimental period being characterized by relatively low ambient temperatures which were insufficient to induce heat stress, resulting in minimal physiological stress associated with heat exposure (28). Likely because the experimental period was characterized by relatively mild ambient temperatures, insufficient to induce substantial heat stress and associated physiological challenges (28). Consequently, oxidative and metabolic challenges were likely diminished, reducing the capacity to detect pronounced effects of antioxidant supplementation (29, 30). Evidence from other studies suggests that the benefits of selenium nanoparticles, particularly plant-derived forms, may become more pronounced under conditions of genuine heat stress, where antioxidant and cellular protective mechanisms are more actively engaged (29–33).

### 4.1 Growth performance under mild environmental conditions

The observed increase in average daily gain of 267 g/day and the lowest feed conversion ratio of 1.98 in CQ-SeNPs supplemented pigs suggest a trend towards improved nutrient utilization (34). Nevertheless, the absence of significant differences (*p* > 0.05) suggests that CQ-SeNPs exerted limited influence under thermoneutral conditions during which the experiments were conducted. Previous research findings have consistently demonstrated that selenium supplementation is most effective under stress conditions such as elevated temperature, oxidative load, or disease challenge, when the redox balance and cellular homeostasis are compromised (35–37). For instance, nano-selenium was reported to alleviate heat stress induced oxidative damage and improved growth performance in broilers by Huang et al. (38). Similarly, SeNPs were found to improve feed efficiency and the immune status of broilers under environmental stress by Debata et al. (39). Under low stress conditions however, the endogenous antioxidant capacities of animals may suffice, rendering additional selenium supplementation less impactful (40). These findings support the view that CQ-SeNPs might show stronger benefits when animals experience more pronounced environmental or oxidative stress. Additionally, age, body condition, breed, and productive status may modulate growth responses, influencing variability in performance under mild or severe heat stress conditions.

### 4.2 Antioxidant enzyme activity and redox regulation

According to our observations, CQ-SeNPs and Na₂SeO₃ supplementation modulated activities of key antioxidant enzymes (CAT, SOD and GPx), though not statistically significant. At 15 days, CQ-SeNPs supplemented pigs displayed numerically higher GPx and GSH/GSSG ratio compared with the Na₂SeO₃ and C groups, consistent with an early enhancement of antioxidant defense, these results are similar to findings by Horky et al. (41). By day 30, CAT activity increased markedly in the Na₂SeO₃ supplemented group, while GPx activity declined across all treatments, suggesting the antioxidant enzymes differential adaptation was temporal, these results are similar to findings on weaned piglets by Qiao et al. (37). This fluctuation likely reflects the sequential activation and regulation of enzymatic defenses, where CAT responds to persistent H₂O₂ accumulation and GPx activity is modulated by substrate availability, age-related enzyme expression and redox demands (35, 42). The CQ-SeNPs group maintained a relatively lower but stable GSH/GSSG ratio, indicating a sustained redox balance (43, 44).

Our findings align with reports that SeNPs enhance antioxidant capacities in tissues through improved bioavailability and incorporation into selenoproteins such as GPx and thioredoxin reductases (45, 46). Notably, phytochemicals from CQ including quercetin, resveratrol analogues, and β-sitosterol exhibit intrinsic antioxidant activities as observed in a study on rat serum by Dadge et al. (47). This suggests that their interaction with selenium may yield synergistic effects, where selenium supports enzymatic ROS detoxification, while CQ polyphenols scavenge free radicals directly or upregulate endogenous antioxidant gene expression (14, 19, 48). CQ-SeNPs likely contributes to redox maintenance through nutritional and phytochemical mechanisms, though this could not be statistically substantiated in our findings. Previous studies show that such synergistic SeNP–phytochemical interactions become substantially more relevant under oxidative pressure, meaning CQ-SeNPs may provide superior protection during heat-induced redox imbalance compared with inorganic selenium (33, 38, 49).

### 4.3 Cellular stress markers

The measurements of iNOS, HSP-70 and 8-OHdG provided additional insight into oxidative and cellular stress responses in the pigs. At 30 days, we observed that Na₂SeO₃ had the highest values for iNOS followed by CQ-SeNPs suggesting that Na₂SeO₃ induced a stronger inflammatory or oxidative response compared to the CQ-SeNPs supplemented group (50). Pigs supplemented with CQ-SeNPs displayed the lowest 8-OHdG levels compared to the Na₂SeO₃ and C groups at 30 days, suggesting reduced nitric oxide production and DNA oxidation (37, 51). Moreover, CQ-SeNPs maintained lower HSP-70 expression, indicating a more stable cellular environment (32, 52). Under actual heat stress, SeNPs including plant-based forms have been shown to strongly suppress HSP-70, reduce oxidative DNA damage, and stabilize nitric oxide production more effectively than inorganic selenium sources (53, 54). This suggests that CQ-SeNPs could yield more pronounced cytoprotective effects in pigs exposed to thermal stress (55, 56).

Our observations support prior studies were selenium nanoparticles mitigated oxidative and inflammatory responses in stressed animals [Liu et al. (32); Zhu et al. (33)]. These findings also highlight that, even under non-stressful temperatures, CQ-SeNPs may enhance baseline antioxidant readiness, potentially improving resilience during sudden environmental fluctuations.

### 4.4 Implications for climate resilience

From a broader perspective, improved antioxidant efficiency and cellular protection contributes to climate resilience in livestock systems (57, 58). Climate variability such as heat and cold stress induces oxidative damage, inflammation and metabolic inefficiency in pigs of all growth stages (58–60). Nutritional strategies that enhance capacities of animals to adapt are therefore integral to sustainable animal production (58). Although this study was conducted under moderate conditions for weaner pigs, the trends observed with CQ-SeNPs although not statistically significant suggest potential benefits under more severe thermal or oxidative stress.

Based on our findings, CQ-SeNPs combined the redox benefits of biogenic selenium with the phytochemical bioactivity of CQ, which may extend protection beyond conventional inorganic Na₂SeO₃ forms (17, 19, 22, 35). Emerging evidence supports that phytogenic selenium nanoparticles improve thermotolerance, mitochondrial stability, and reduce systemic inflammation under heat stress, indicating that CQ-SeNPs could be valuable for climate-resilient pig production (15, 17, 22, 39, 61, 62).

### 4.5 Limitations and future perspectives

The main limitation to this study is the absence of a defined stress challenge. Under moderate temperatures, baseline oxidative stress was likely minimal, limiting the observable effects of supplementation, leading to small treatment effects. Variability related to age, body condition, and breed may have influenced physiological responses, contributing to wide ranges in enzyme and stress-marker values. Future work should evaluate CQ-SeNPs under controlled thermal stress to better assess their potential for mitigating heat-induced oxidative damage. Examining tissue selenium distribution, selenoprotein transcription, and inflammatory cytokines would further elucidate mechanisms of action. Studies with larger sample sizes, varied dosages, and extended durations could optimize practical supplementation strategies. Given current climate trends, understanding how CQ-SeNPs support resilience during heat waves is particularly relevant for pig production in tropical regions, including Zambia.

## 5 Conclusion

Under moderate temperatures, non-stressful environmental conditions, dietary supplementation with CQ-SeNPs resulted in modest but non-significant improvements in growth performance. These findings suggest that CQ-SeNPs can maintain redox balance and reduce oxidative damage even in the absence of thermal stress, potentially enhancing physiological preparedness for future challenges such as heat stress. Although the effects were modest, the synergistic antioxidant potential of selenium and *C. quadrangularis* phytochemicals represents a promising strategy to improve resilience in porcine production. Further research under heat stress conditions and on dose–response is needed to confirm these findings and to establish CQ-SeNPs as a viable nutritional strategy for climate adaptation and sustainable livestock production.

## Data Availability

The raw data supporting the conclusions of this article will be made available by the authors, without undue reservation.
